# Constrained transformer network for ECG signal processing and arrhythmia classification

**DOI:** 10.1186/s12911-021-01546-2

**Published:** 2021-06-09

**Authors:** Chao Che, Peiliang Zhang, Min Zhu, Yue Qu, Bo Jin

**Affiliations:** 1 Key Laboratory of Advanced Design and Intelligent Computing, Ministry of Education, Dalian University, Dalian, 116622 China; 2 Zhejiang Institute for Quality Inspection of Light Industrial Products, Hangzhou, 310000 China; 3 School of Innovation and Entrepreneurship, Dalian University of Technology, Dalian, 116024 China

**Keywords:** ECG signal, CNNs, Transformer, Link constraints

## Abstract

**Background:**

Heart disease diagnosis is a challenging task and it is important to explore useful information from the massive amount of electrocardiogram (ECG) records of patients. The high-precision diagnostic identification of ECG can save clinicians and cardiologists considerable time while helping reduce the possibility of misdiagnosis at the same time.Currently, some deep learning-based methods can effectively perform feature selection and classification prediction, reducing the consumption of manpower.

**Methods:**

In this work, an end-to-end deep learning framework based on convolutional neural network (CNN) is proposed for ECG signal processing and arrhythmia classification. In the framework, a transformer network is embedded in CNN to capture the temporal information of ECG signals and a new link constraint is introduced to the loss function to enhance the classification ability of the embedding vector.

**Results:**

To evaluate the proposed method, extensive experiments based on real-world data were conducted. Experimental results show that the proposed model achieve better performance than most baselines. The experiment results also proved that the transformer network pays more attention to the temporal continuity of the data and captures the hidden deep features of the data well. The link constraint strengthens the constraint on the embedded features and effectively suppresses the effect of data imbalance on the results.

**Conclusions:**

In this paper, an end-to-end model is used to process ECG signal and classify arrhythmia. The model combine CNN and Transformer network to extract temporal information in ECG signal and is capable of performing arrhythmia classification with acceptable accuracy. The model can help cardiologists perform assisted diagnosis of heart disease and improve the efficiency of healthcare delivery.

## Background

Heart disease is one of the most pervasive causes of human death [1]. An electrocardiogram (ECG) is a technique for graphical representation of heart activity over time. An ECG reflects the regularity of the heart’s activity and physiological state of each body part. Therefore, an ECG is a significant reference for the diagnosis of heart disease [2]. The difficulties in diagnosing heart disease are mainly related to its paroxysmal and complex nature. In a clinical manner, doctors usually diagnose it based on the morphological waveform of an ECG, although it is usually difficult to make a clinical judgment, especially when the signals are mixed with noise. This stresses the significance of developing methods to accurately identify heart disease with support from machine learning.

In the past few decades, many machine learning methods have been employed to perform intelligent analysis of ECG signals. Given the morphological characteristics of an ECG waveform, such as the shape of the QRS and P waveforms, traditional machine learning approaches usually employ fixed features and classical signal processing techniques [3, 4]. However, even the same patient exhibits different waveforms in different environments. Therefore, using fixed features are not sufficient to accurately distinguish different types of diseases [5, 6]. Moreover, most of the existing models require manually crafted features. In these cases, the selection of features for the inputs would significantly affect the performance of implemented classifiers.

In recent years, end-to-end deep-learning methods have led to substantial breakthroughs in image classification, speech recognition, and other tasks. It is also a significant research problem to effectively apply related techniques in the fields of medicine and healthcare. Cao et al. combined Brownian multi-verse optimizer (BMVO) algorithm [7] and a Damping Multi-Verse Optimizer (DMVO) algorithm [8] with DNA storage to show us how closely future disease prediction is linked to DNA storage. Recently, deep-learning methods have been applied for ECG signal processing and heart disease diagnosis. Research on ECG signals has traditionally been a hot research topic. Here, the general framework of ECG diagnosis is introduced, and then previous work on automatic ECG diagnosis in the literature is reviewed. Earlier, large, rich ECG datasets were not available. Therefore, it is not particularly time-consuming to capture ECG features manually, such as the QRS wave group and S and T waves. Chazal et al. proposed an algorithm for personalized heartbeat classification based on ECG morphology and discriminant analysis using time-interval feature linearity [9]. In 2004, Chazal et al. proposed a method for automatically processing heartbeat classification, which divides the manually detected heartbeat into six categories: normal pulsation, ventricular ectopic beat (VEB), supraventricular ectopic beat (SVEB), normal and VEB fusion, and unknown beat type. A statistical classification model with a supervised method has been used, but the detection effect on the SVEB type is relatively weak [10]. Varatharajan et al. performed pre-processing of some filtering, such as FIR and IIR, on ECG signals, and the filtered signals were input into an improved support vector machine (SVM) for pattern recognition through a linear discriminator [11]. Shadmand also proposed an artificial neural network based on particle swarm optimization to classify specific patient heartbeats. Compared to the above algorithms, Shadmand’s algorithm has superior classification performance [12]. All of these methods use manual features to train models and only achieve limited performance. Thus, they cannot help doctors play a supporting role.

In 2010, Zhang et al. constructed an ECG database, the China Cardiovascular Disease Database (CCDD), including both 12-lead ECG and detailed diagnostic data. It contains more than 190,000 12-lead ECG records and each record has at least one tag [13]. Using this database, Jin and Dong proposed a CNN model and designed a three-layer convolution layer followed by the fully connected layer. The model reached an accuracy of 83.66% on classification experiment [14]. Recently, a research team at Stanford University (California, USA) developed a deep neural network (DNN) to classify a broad range of distinct arrhythmias from single-lead ECGs with high diagnostic performance, which outperformed the diagnoses of cardiologists [15]. Shashiku et al. designed a convolutional de-noising autoencoders model to identify ECG heartbeat classifications [16]. Jun et al. used a remote ECG database to create a new dataset and proposed an end-to-end deep CNN to identify short-term 12-lead ECG signals. They improved the residual module, which is more expressive than a doctor’s judgment for disease identification in six categories [17]. Yao et al. proposed attention-based time-incremental convolutional neural network (ATI-CNN) [18], a deep neural network model achieving both spatial and temporal fusion of information from ECG signals by integrating CNN, recurrent cells and attention module. The above algorithms all use CNNs to identify the types of ECG signals [19, 20].

Although CNNs have achieved great success in the recognition of arrhythmias, at the same time, the ECG signal is also a time series of data, and a recurrent neural network (RNN) can be used to solve the time-series problem. In recent years, for example, Mostayedl et al. used a two-way RNN to identify multiple categories of arrhythmia. He pre-processed the ECG signal to obtain characteristic information of ECG signals such as the positions of the R peak and of the QRS complex. The information of these features is then input into the bidirectional RNN model to obtain the identification classification of ECG signals [21]. For example, Saadatnejad et al. designed both a class of network-recognition ECGs based on the wavelet transform and multiple Long Short-Term Memory (LSTM) models for ECG signals of personal wearable devices [22]. Considering the specificity of ECG signals, Chen et al. completed the arrhythmias classification by fusing CNN and RNN models with excellent performance on the dataset we used [23].

The most representative work was reported by Hannun et al. [24]. They collected single-lead ECG data from wearable displays and used a 34-layer residual CNN to diagnose the signals. The approach demonstrated high diagnostic performance, even exceeding the average level of cardiologists in F1 score. However, it ignored the characteristics of ECG as temporal signals. A transformer network can capture temporal features and focus on context vectors using an attention mechanism [17, 22]. To this end, an end-to-end deep-learning model that can effectively process arbitrary-length 12-lead ECG signal sequences by extending the transformer model is proposed herein. Specifically, the model splits an ECG record into different segments using a window of 6-s duration, which are used as inputs of the model, and then it captures the valuable features by a CNN and feeds them into the transformer network. The transformer network employs a multi-head attention mechanism to pay more attention to different segments of ECG signals. Importantly, a new constraint for classifying the ECG signals has been designed, which leads to the prior knowledge, i.e., the labels of ECG signals. The main role of the link constraint is to make the embedding vectors of the ECG signals from the same class as similar as possible. Owing to the extraction of valid and significant features, the link constraint can result in enhanced performance for the downstream tasks.

Overall, the main contribution of this paper are the following.

- A transformer network is embedded in the CNN framework for the identification of ECG signals. The integration of the transformer compensates for the shortcomings of the CNN for poor performance of temporal features.
- A new link constraint is introduced in the loss function. In the previous schemes, the embedding features were not evaluated; however, the proposed model constrained the features with the new link constraints to ensure that the network extracts better feature information.
- The time window was designed to process unequal ECG signals so as not to lose the temporal information of the signal.

The rest of this paper is organized as follows. In Section 2, related work is summarized. In Section 3, a formal problem statement is provided and the data pre-processing issues are introduced. The proposed methodology is discussed in Section 4. The experimental settings are discussed in Section 5, and results are presented. Finally, conclusions are drawn in Section 6.

## Methods

### Problem statement and data processing

Before discussing methodology, the diagnosis problem that is the aim of this paper is clarified and the data characteristics discussed.

#### Problem statement

**Fig. 1:**
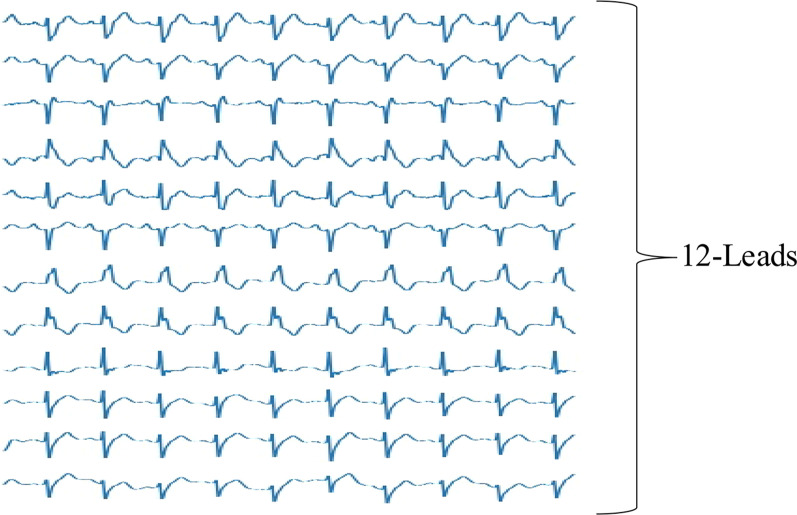
Twelve-lead ECG sample

ECG data used in this paper were acquired from a cardiology challenge [25], which was collected from 11 hospitals and covering a total of 6877 individuals. These data have been de-sensitized, with a total of 3699 records for men and 3178 for women. The duration of the signal is between 6 and 60 s, with an average duration of 15.79 s. The data were recorded by a 12-lead ECG, with a frequency of the electrocardiogram recording of 500 Hz. The 12 waveforms of an ECG signal sample are presented in Fig. 1.

Each sample has a tag (label) for its category. There are nine categories in total, including one normal type of heart disease and eight abnormal types.The data category description are shown in Table 1. The main problem to be solved in this work can be formulated as follows: given 12-lead ECG signal data, the data are segmented through a time window and fed into a model for learning, and finally the classification scores of the 9 categories are obtained using the classification model.

**Table 1:** Data category description

Type	Description
Atrial fibrillation (AF)	Atrial fibrillation (AF) is characterized by the fibrillatory atrial waves and irregular conduction of QRS
First-degree atrioventricular block (I-AVB)	First-degree atrioventricular block (I-AVB) is defined as constant PR intervals longer than 0.2 s
Left bundle branch block (LBBB)	Left bundle branch block (LBBB) is diagnosed by the distinct QRS morphology at leads I, aVL, V1, V2, V5, and V6
Right bundle branch block (RBBB)	Right bundle branch block (RBBB) is diagnosed by the rsR0pattern at V1 and V2
Premature atrial contraction (PAC)	The premature atrial contraction (PAC) indicate the electrical impulse from an abnormal site; specifically, the P wave or QRS morphology of PAC differs from that in normal heart beats
Premature ventricular contraction (PVC)	The premature ventricular contraction (PVC) indicate the electrical impulse from an abnormal site; specifically, the P wave or QRS morphology of PVC differs from that in normal heart beats
ST-segment depression (STD)	ST segment is abnormal if either ST-segment depression (STD) is greater than 0.1 mV
ST-segment elevated (STE)	ST segment is abnormal if either ST-segment elevation (STE) is greater than 0.1 mV

#### Data pre-processing

Noise is inevitable in collecting ECG signals. Noise includes baseline drift and high-frequency noise. There are many ways to de-noise ECG signals, such as designing high-pass or median filters to eliminate baseline drift. In this paper, we apply the difference method and wavelet transform in signal processing to improve the quality of the signal. For the abnormal values that appear in the ECG signals, it is found that the abnormal values have relatively larger voltage values than the normal signals, so we use the difference method to remove these abnormal values. First, we set the threshold values after traversing the complete ECG signals, and replace the abnormal values with the threshold value when the voltage values is greater than the threshold values. Then, we can obtain ECG data with no abnormal values. For the ECG signals containing noise, we performed six layers of wavelet decomposition on the ECG signals and selected the bior2.6 wavelet function to obtain the detail coefficients and approximation coefficients of each layer. The EMG interference noise is distributed in the high-frequency components of the first layers of decomposition, while the noise of baseline offset is distributed in the low-frequency components of the sixth layer. Therefore, we set all the detail coefficient components in the first and second layers to 0, and set the approximate coefficient components in the sixth layer to 0. Finally, we reconstruct the signal layer by layer. After the reconstruction, we obtain the ECG signal without outliers and noise. The combination of difference method and wavelet transform method can eliminate noise interference and outliers.

Since the length of the ECG signals is not equal, we split the ECG signals into segments of fixed length according to the given window size and step size. The size of each window is set to match the integrity of the regular heartbeat. All experimental parameters will be given in “Experimental settings” section.

### Model architecture

**Fig. 2:**
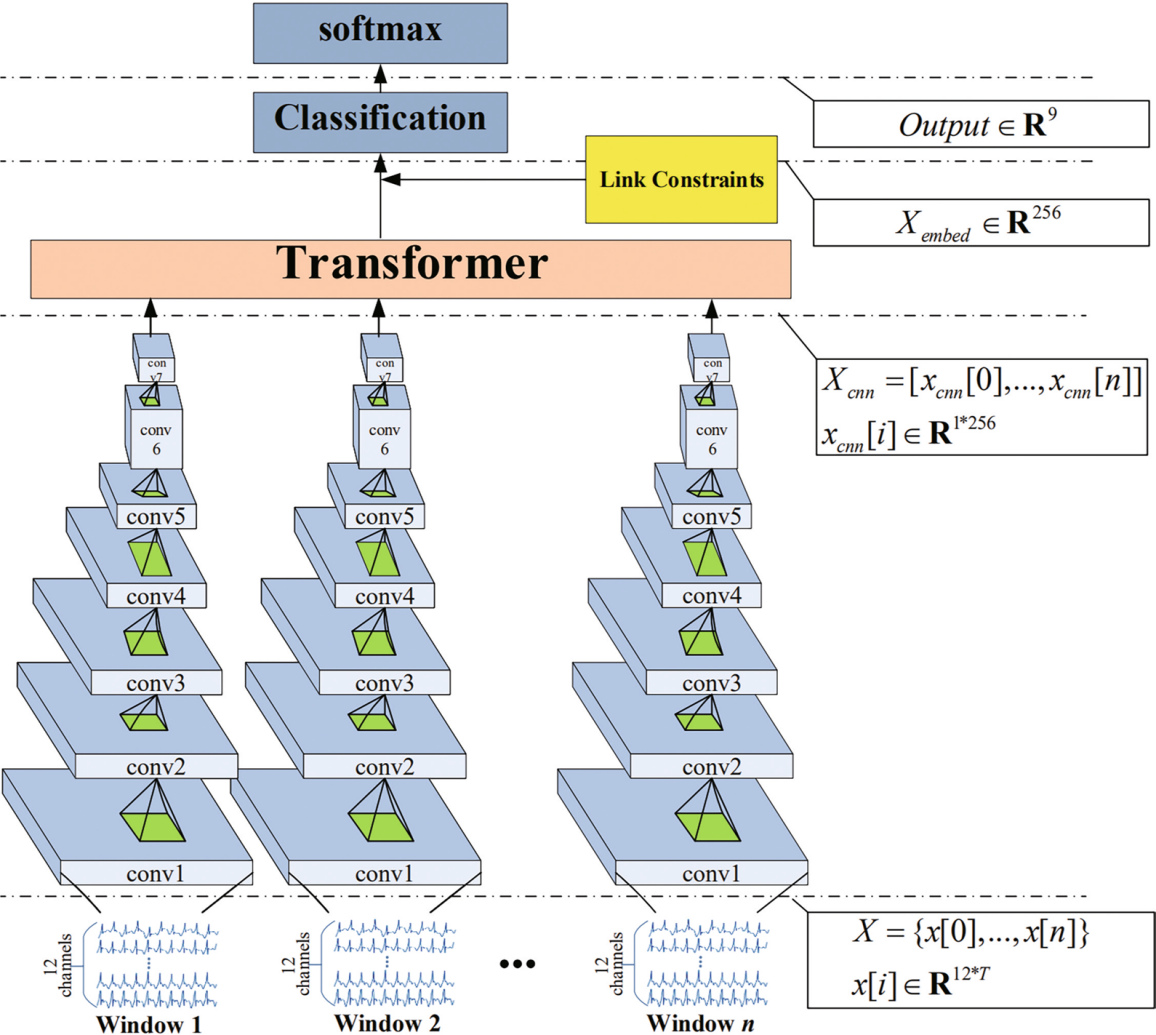
Architecture of proposed model

A new end-to-end model for ECG classification was designed that combines the advantages of a CNN and transformer networks. The architecture of the proposed model, which is designed to handle variable-length 12-leads ECG data, is shown in Fig. 2. An ECG record is divided into equal-length ECG signal segments according to the window size and step size given in the pre-processing stage. The 12-lead data are then passed to the CNN to capture the hidden deep features in the ECG signal.

Next, the linear network structure is used to further capture the feature information, which was then sent to the transformer network in the form $X_{cnn}=[x[0],x[1],...,x[n]]$. The transformer network can output the embedding vector of the input ECG signal $X_{embed}$, which is finally fed into the classification layer to obtain the class probability of the ECG signal.

As shown in Fig. 2, the proposed model consists of four components: (1) the link constraints, (2) feature-extraction units, (3) transformer network, and (4) classification layers.

#### Link constraints

To improve the quality of embedding features for downstream task, the following assumption on the embedding features is made.

If the correlation coefficient between embedding features of two samples are large ($max=1$), which means positive correlation, the classifier will predict that they belong to the same category with a high probability. If the correlation coefficient is small ($min=-1$), the classifier will predict different categories with a high probability.

Based on the above assumptions, the correlation coefficient between the samples of the same class is made a larger value by $\min \left\| X_{embed}^i-X_{embed}^j\right\| _2^2$. In contrast, the correlation coefficient between the samples of different classes is made smaller by $\min \left\| X_{embed}^i+X_{embed}^j\right\| _2^2$. In the extreme condition, $X_{embed}^i=X_{embed}^j$ when the correlation coefficient equals 1, and $X_{embed}^i=-1 X_{embed}^j$ when the correlation coefficient equals −1.

**Fig. 3:**
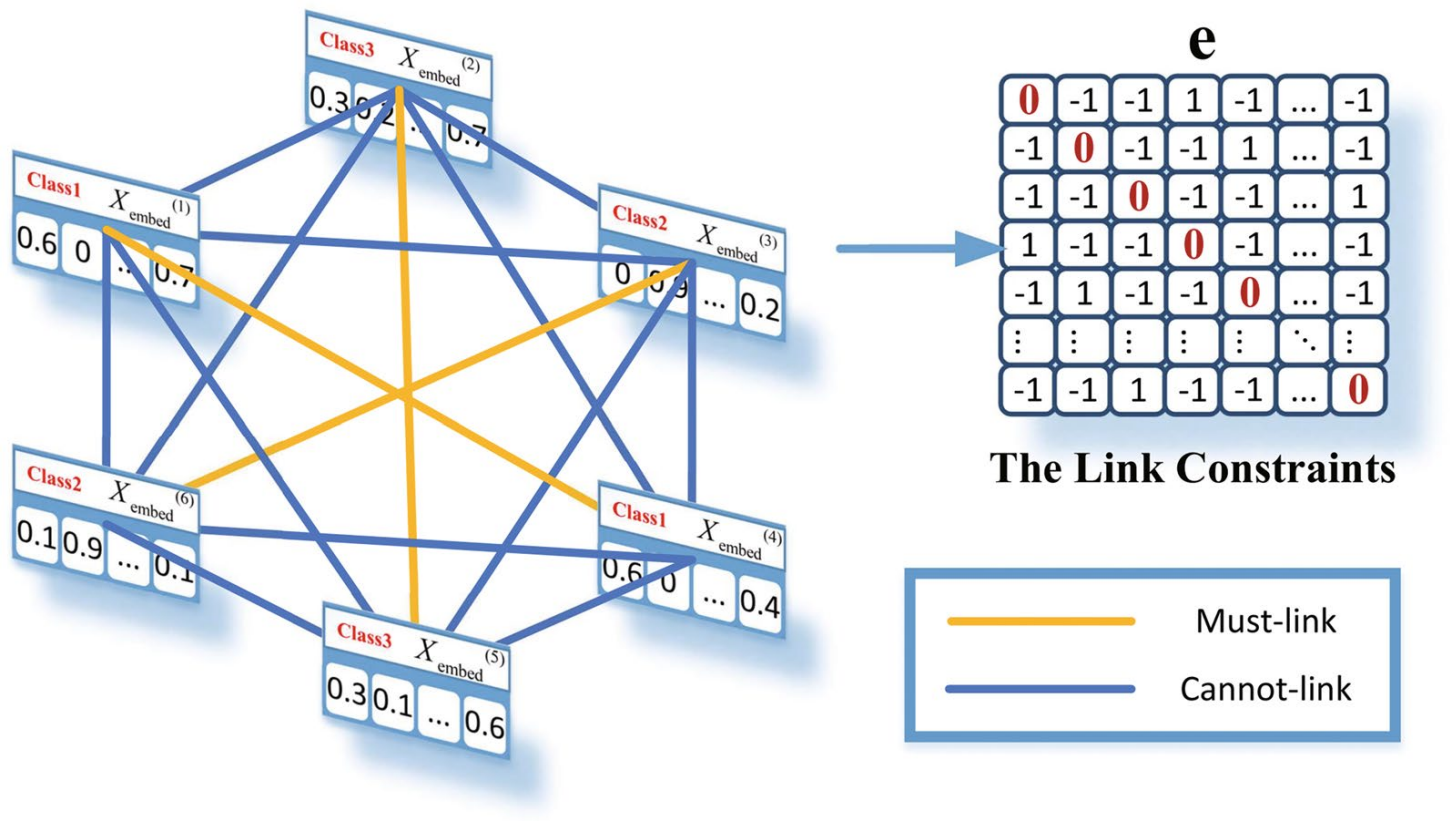
Schematic diagram of link constraints

Borrowing the idea of [26], link constraints are added to the loss function. There are two types of links between the samples: a Must-link and a No-link. For the task of classification, the links between the samples of the same class are Must-links and the links between the samples of the different classes are No-links. Figure 3 shows that the embedding vectors of two samples are similar when they have a Must-link. Thus, the embedding vectors can better contribute to downstream tasks such as classification. It is essentially a regular term, and its formula is:

1 $$\begin{aligned} \begin{aligned} Loss = \min {f(\beta )+\lambda I(\beta )} \end{aligned}\end{aligned}$$

2 $$\begin{aligned} \begin{aligned} I(\beta ) =\sum _{pq}\frac{1}{2}\left\| \beta ^{p}-e_{(pq)} \beta ^{(q)}\right\| _{2}^{2} \end{aligned}\end{aligned}$$

3 $$\begin{aligned} \begin{aligned} e_{(ij)}= {\left\{ \begin{array}{ll} 1 &{} \hbox {A must-link between} i\,hbox{and}\,j \\ -1 &{} \hbox {A cannot-link between} i\,\hbox {and}\,j \end{array}\right. } \end{aligned} \end{aligned}$$

Based on Eqs. (1)–(3), the link constraints can make the embedding vectors of the same class closer and those of different classes. In the experiment, the embedding vector $\beta$ is $X_{embed}$ and the function *f* the classifier network (a linear network) after the transformer network. Moreover, the link constraints can only be applied in the training process like other regularization terms, such as L1 and L2. The specific process of link constraints is detailed in Algorithm 1.

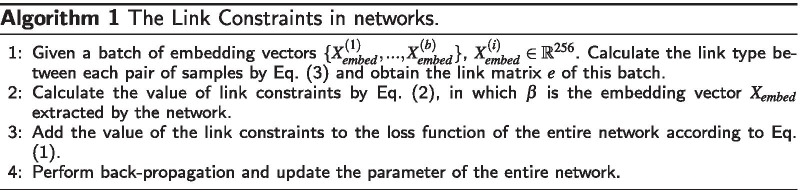



Although the outputs $X_{cnn}$ of the CNN [27], such as $\beta$ can be used, since $X_{cnn}$ sometimes has temporal information, i.e., the first element may have the information from the early time and the last element may have the late-time information, we cannot use outputs from a CNN as embedding vectors directly. Therefore, several layers are needed to disorganize the temporal information and usually take the outputs of BiLSTM or a transformer as the embedding vectors.

#### Feature extraction

**Fig. 4:**
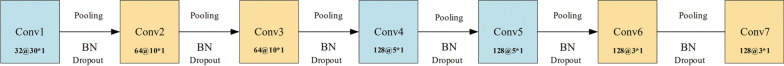
CNN layer parameters

CNNs have shown outstanding performance in image-classification tasks due to their translation-invariance and ability to capture local features [28, 29]. The essence of the convolution kernel is a filter, which is especially suitable for feature extraction of ECG signals. A CNN network with seven convolution layers, which have different kernel sizes to capture various features, was designed in the present study. Each convolution layer is composed of a convolution filter, batch normalization layer [30], active layer, and pooling layer. The parameters of the CNN’s layers are shown in Fig. 4.

**Fig. 5:**
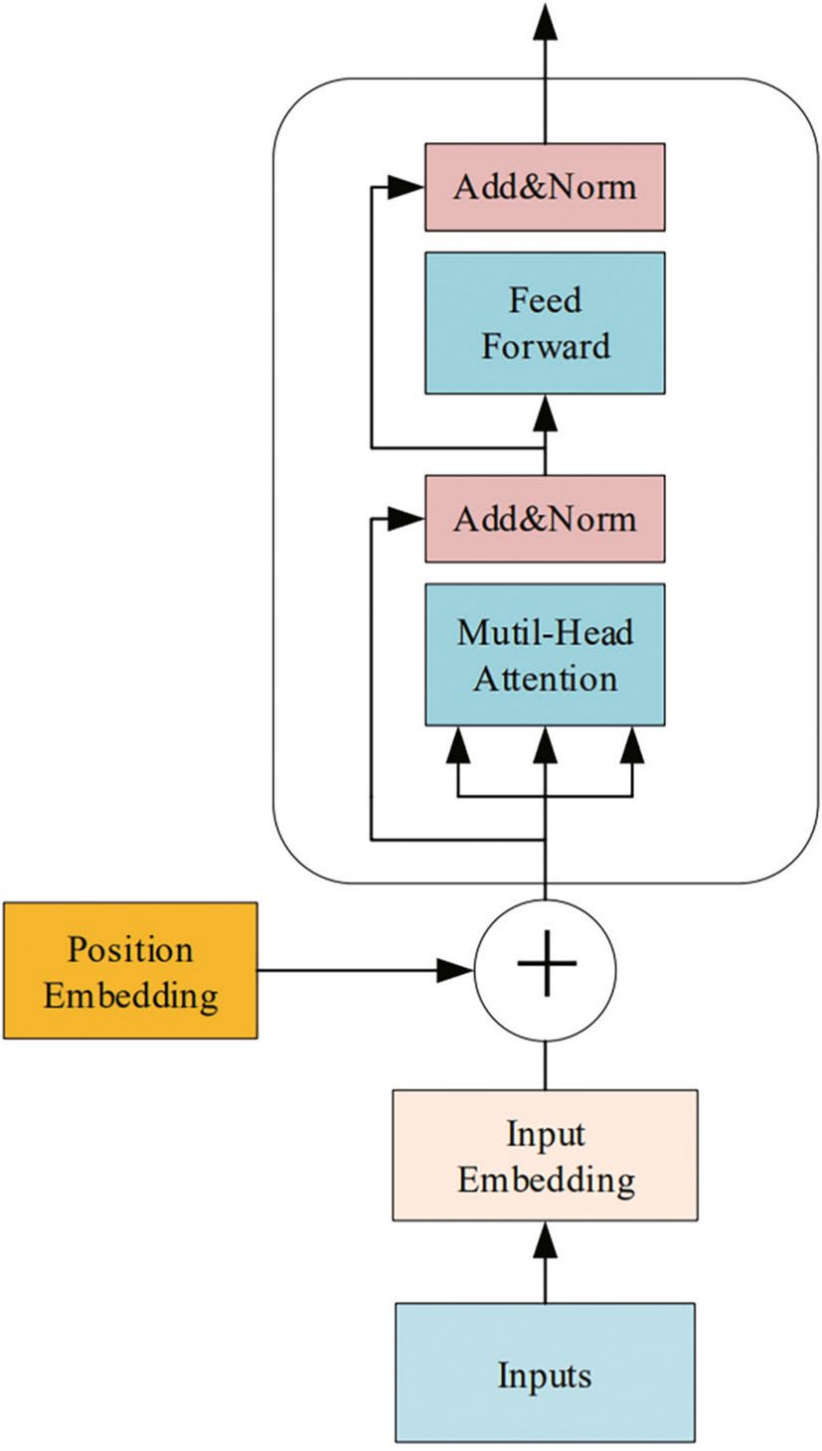
Structure of transformer-network encoder

#### Transformer layers

The transformer network [31] was developed based on the attention mechanism, which is composed of an encoder and decoder. In the ECG signal classification problem, only the encoder part is used, the structure of which is shown in Fig. 5. The transformer network contains eight identical layer stacks and each layer has two sub-layers. The first sub-layer is the multi-head attention and the second is a simple fully connected forward neural network. The two sub-layers are connected by a residual network structure followed by a norm layer. The output of each sub-layer can be expressed by $out = LayerNorm(x+Sublayer(x))$, where each sub-layer is constructed independently. To facilitate the residual connection between layers, the sub-layers in the model are fixed output with 256 dimensions. These sub-layers are described as follows.

- Scaled dot-product attention. The input of the attention function *Q*, *K*, and *V* represents query, key, and value, respectively. The attention weight is calculated according to the similarity of the query key. The attention context is obtained according to the attention weights. The model uses scaled dot-product attention, which is calculated as follows:
  4 $$\begin{aligned} \begin{aligned} Attention&(Q,K,V) = softmax(Q K_{T}/\sqrt{d_{k}} V) \end{aligned} \end{aligned}$$
- Multi-head attention. The multi-head attention mechanism projects *Q*, *K*, and *V* through *h* different linear transformations, and finally splices different attention results. *Q*, *K*, and *V* have the same values in the self-attention mechanism. The formula is expressed as follows:
  5 $$\begin{aligned} \begin{aligned} MultiHead&(Q, K, V) = Concat(head_{1},...,head_{h}) \end{aligned} \end{aligned}$$
  6 $$\begin{aligned} \begin{aligned} head_{i} =Attention(QW_{i}^{Q},KW_{i}^{K},VW_{i}^{V}) \end{aligned} \end{aligned}$$
  where *MultiHead*(*Q*, *K*, *V*) is the contact of $head_{i}$.
- Position-wise feed-forward networks. In addition to the attention sub-layer, each layer of the encoder contains a fully connected feed-forward network and a two-layer linear transformation using a ReLU activation function:
  7 $$\begin{aligned} \begin{aligned} FFN(x)= max(0,xW_{1}+b_{1})W_{2}+b_{2} \end{aligned} \end{aligned}$$
  While the linear transformations are the same across different positions, they use different parameters from layer to layer. The input size of the model is 256 and the size of the hidden layer is 1024.
- Positional encoding. To make use of the order of sequence, “position encoding,” i.e., the relative or absolute position of the sequence, is added to the input embedding at the top of encoder. The positional encoding (PE) dimension is $d_{model=256}$, the same as input embedding:
  8 $$\begin{aligned} \begin{aligned} PE_{(pos,2i)}=sin(pos/10000^{2i/d_{model}}) \end{aligned} \end{aligned}$$
  9 $$\begin{aligned} \begin{aligned} PE_{(pos,2i+1)}=sin(pos/10000^{2i/d_{model}}) \end{aligned} \end{aligned}$$
  where *pos* is the position and *i* the dimension.

#### Classification layers

The transformer network is connected to the classification layer for multi-classification. The classification layer is composed of linear layers and activation layers. The classification network outputs the probability that each patient may have for each type of heart disease.

## Results

### Experimental settings

The ECG data of 6877 patients were divided into training and test sets in a ratio of 9:1. The experimental parameters are shown in the Table 2. The model is trained using Adam optimizer. A cross-entropy function was employed as the loss function.

**Table 2:** Experimental parameter settings

Experimental parameters	Size	Experimental parameters	Size
ECG window size	3000	Step size	1500
Input size	150	Hidden layer size	1024
Batch size	100	Epoch	150
Learning rate	0.001		

### Evaluation metrics

In medical diagnosis, a lower misdiagnosis rate relative to correct diagnoses is optimal, so F1 score is employed as the evaluation metric. F1 score is the harmonic mean of the positive predictive value and sensitivity, and is computed as follows:

10 $$\begin{aligned} \begin{aligned} F_{1,j}=2 N_{ii}\big / \sum _{j-1}^{9}(N_{i,j}+N_{j,i}) \end{aligned} \end{aligned}$$

where $N_{i,j}$ indicates the number of samples in the *i*th class that are classified into the *j*th class, and $F_{1,j}$ is the value of macro-F1 of the *j*th class.

### Experimental results

#### Trends in accuracy and F1

**Fig. 6:**
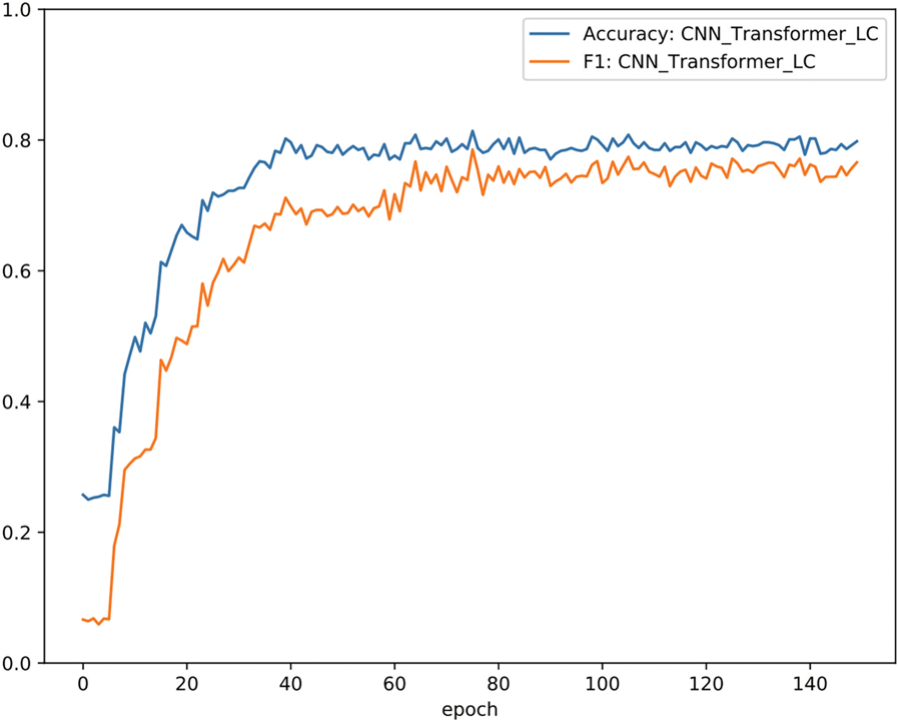
Accuracy and F1 of CNN_Transformer_LC in 150 epochs

Figure 6 illustrates that the training data increase in accuracy and recall as the number of iterations increases. These results show that the model can extract not only effective features in the training data, but also extract the same effective hidden features from the unknown test data for classification. The results verify the generalizability of the proposed algorithm.

#### Comparisons of classification performance

Five SOTA deep-learning models were employed as baselines to compare with CNN_Trans- former_LC: A CNN [32], ResNet [33], Multi_channelCNN [34], BiRNN [21], and CNN_BiLSTM [30]. We applied the methods of these models to the dataset of the paper and obtained experimental results for different models. The experimental results are reported in Table 3. As can be seen from the table, Multi_channelCNN outperforms CNN-based models in multiple disease categories. The experimental results of the CNN_Transformer_LC model in the verification set are better than those of CNN, ResNet, Multi_channelCNN, and BiRNN. As the CNN can only input fixed-length time-series vectors, some feature information will be lost, leading to a performance decrease. RNN inputs all the signal information, so the RNN experimental results are better than those of the CNN. At the same time, the performance of the CNN_Transformer_LC model is better than that of the CNN and BiRNN. The CNN and BiRNN classify the extracted artificial features, while CNN_Transformer_LC can extract more effective feature vectors than artificial features. CNN_Transformer_LC significantly improves the recognition rate of I-AVF, LBBB, and STE.

**Table 3:** Comparison of classification results of CNN_Transformer_LC and baselines on different Arrhythmia classifications

Methods	Normal	AF	I-AVF	LBBB	RBBB	PAC	PVC	STD	STE	F1
CNN	0.578	0.709	0.753	0.773	0.825	0.207	0.376	0.562	0.389	0.574
ResNet	0.578	0.787	0.833	0.757	0.847	0.324	0.407	0.610	0.260	0.601
Mutil_channelCNN	0.666	0.733	0.827	0.8	0.821	0.421	0.648	0.575	0.32	0.646
BiRNN	0.738	0.768	0.742	0.705	0.821	0.59	0.807	0.658	0.294	0.742
CNN_BiLSTM	0.723	0.826	0.851	**0.829**	**0.893**	0.600	0.818	0.692	0.529	0.751
CNN_Transformer_LC	**0.817**	**0.858**	**0.878**	0.800	0.872	**0.618**	**0.830**	**0.711**	**0.686**	**0.786**

Bold values indiate the best experimental results under this category

To investigate the effectiveness of different parts of the proposed model, ablation experiments were conducted, the results of which are reported in Table 4. As can be seen from the table, the CNN_BiLSTM model significantly outperform the CNN. This is because the output of convolutional neural layer is time-serially related in the ECG signal classification problem. After using a RNN to process the relevant output of the CNN, the result obtained is better. This also reflects that the RNN can further extract effective features. Owing to the dependency of ECG signals , the performance of the CNN with the transformer is greatly improved compared with that of a single CNN. This also proves that a transformer can effectively extract such features. However, it is also found in Table 4 that the F1 scores of PAC, STE, and STD are not as good as those of other categories, because the limited data distribution of the three categories in this dataset results in low performance. At the same time, Table 4 shows that using a link constraint can effectively suppress the influence of data imbalance and improve the performance to some extent in the PVC, STD, and STE categories.

**Table 4:** Results of ablation experiments on different Arrhythmia classifications

Methods	Normal	AF	I-AVF	LBBB	RBBB	PAC	PVC	STD	STE	F1
CNN	0.578	0.709	0.753	0.773	0.825	0.207	0.376	0.562	0.389	0.574
CNN_BiLSTM	0.723	0.826	0.851	**0.829**	**0.893**	0.600	0.818	0.692	0.529	0.751
CNN_BiLSTM_Attention	0.739	**0.867**	0.851	**0.829**	0.889	0.617	0.823	0.679	0.538	0.759
CNN_BiLSTM_LC	0.794	0.857	0.894	0.722	0.870	**0.672**	0.787	0.688	**0.700**	0.776
CNN_Transformer	0.810	0.855	**0.912**	0.769	0.873	0.635	0.750	0.704	0.571	0.764
CNN_Transformer_LC	**0.817**	0.858	0.878	0.800	0.872	0.618	**0.830**	**0.711**	0.686	**0.786**

Bold values indiate the best experimental results under this category

#### Visualization of embedding vectors

**Fig. 7:**
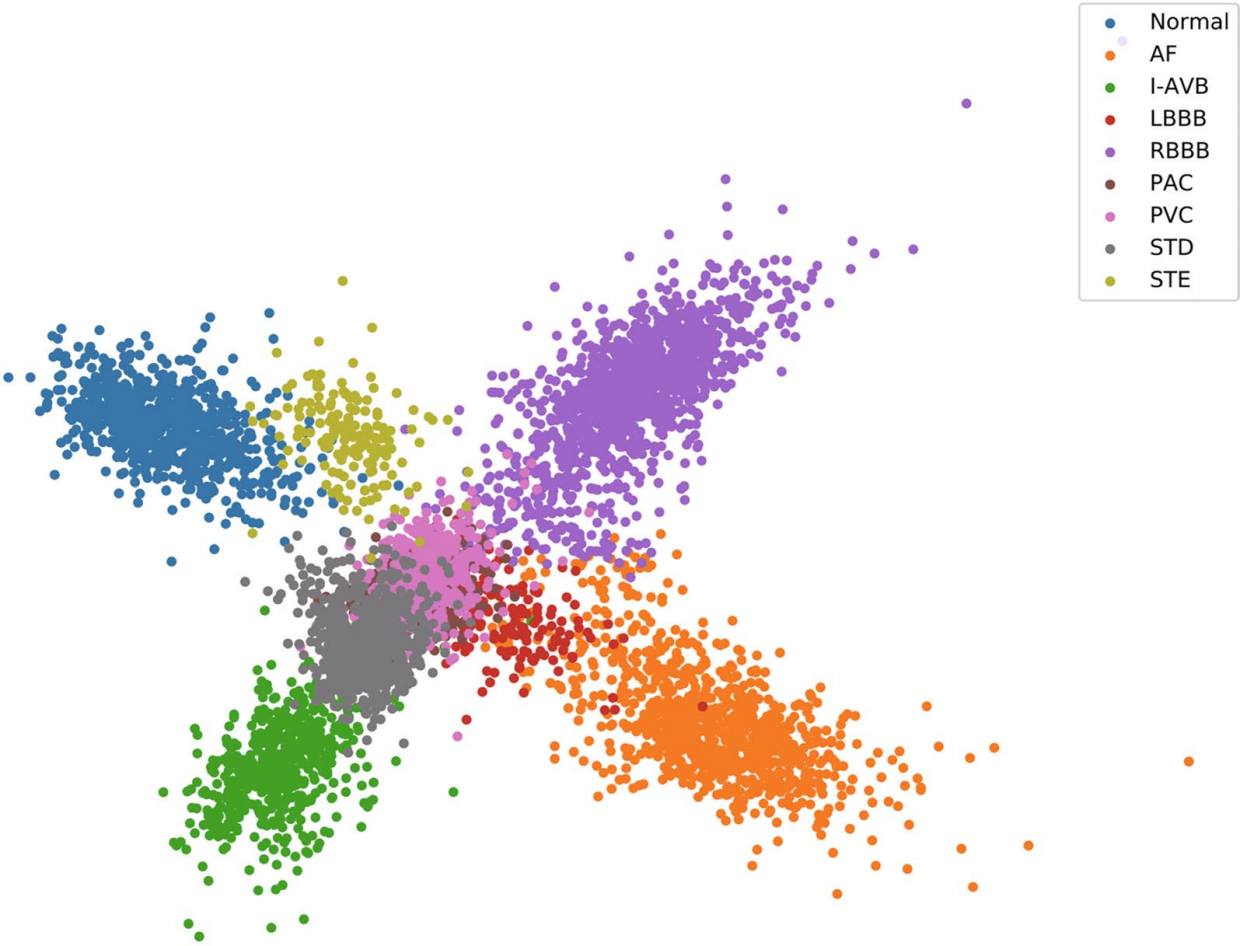
Embedding vectors of nine classes of heart disease

To confirm the ability of feature extraction of the proposed model, dimensionality reduction was performed on the embedding vectors using principal components analysis (PCA) and the results visualized in Fig. 7. It can be seen that the embedding representation obtained by the proposed model can effectively separate most categories. Because of the link constraints, the samples belonging to the same categories are close to each other and the samples belonging to different categories are far from each other, which can help distinguish the vectors in different categories.

#### Embedding similarity matrix

To demonstrate the effect more concretely, center vectors $X_{class\ i},\ i=1,2,...,9$, are defined for each class, which are the closest vectors of each embedding vector in all classes in the training set. The nine vectors $X_{class\ i},\ i=1,2,...,9$, are calculated as follows.

11 $$\begin{aligned} \begin{aligned} X_{class\ i} = \underset{x}{{\text {argmin}}} \sum {\Vert x - X_{embed}^{h}\Vert _{2}^{2}}, \quad sample^h \in class\ i \end{aligned} \end{aligned}$$

**Fig. 8:**
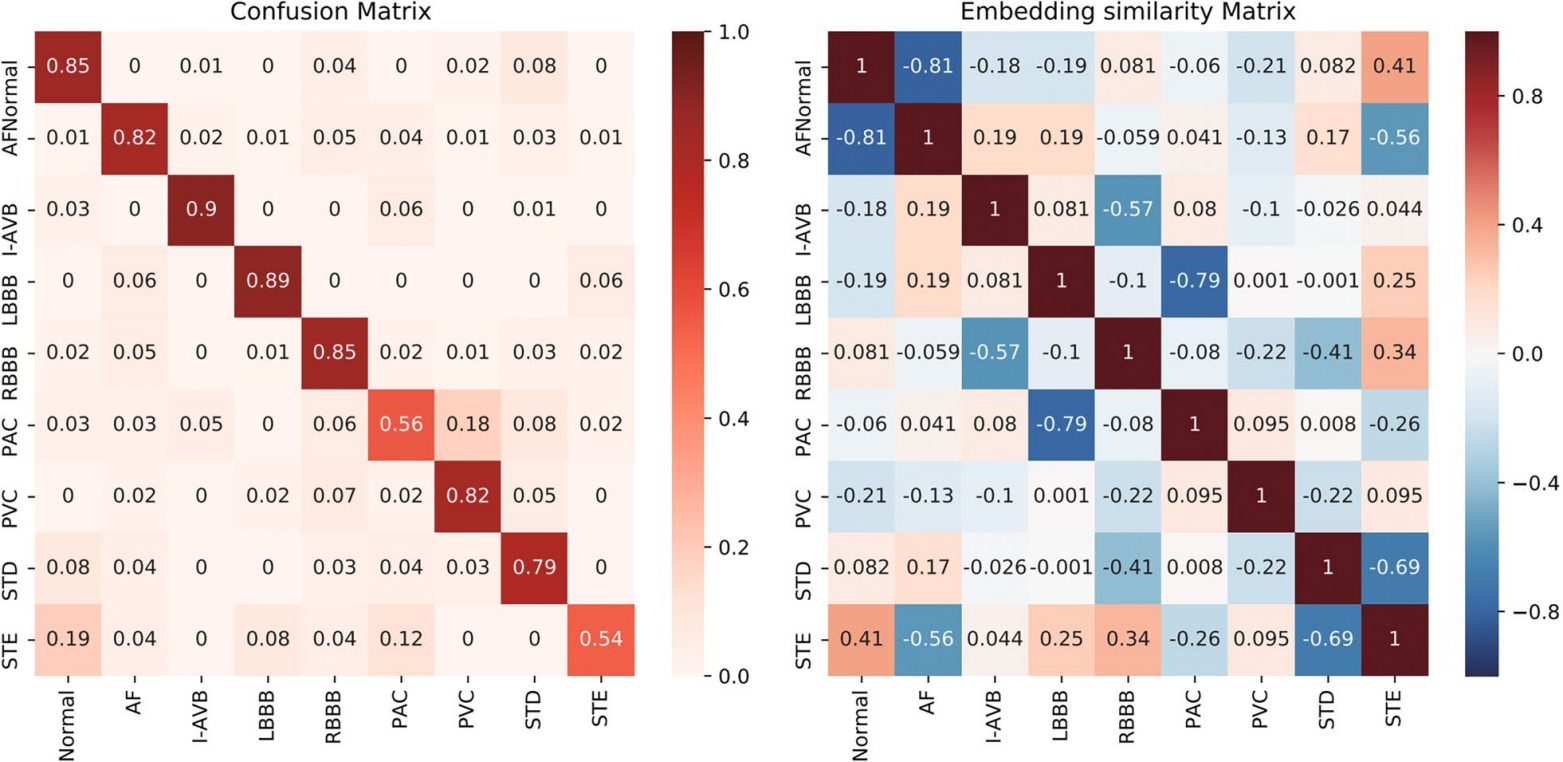
Confusion matrix and embedding similarity matrix

Then, the similarity matrix of the nine vectors is obtained by the Pearson correlation coefficient. Figure 8 shows the confusion matrix of the proposed method in the testing set and the embedding similarity matrix in the training set. An interesting rule is found from the confusion matrix, namely, if classes *i* and *j* have a large similarity value (correlation coefficient), the examples in classes *i* or *j* have a high probability of being classified into other classes. The smaller the similarity values between two classes, the less likely it is that the instances of two classes will be misclassified. If two classes have negative similarity, almost no misclassification occurs between them. This is the same as the proposed assumption in “” section, which confirms the correctness of using link constraints.

**Fig. 9:**
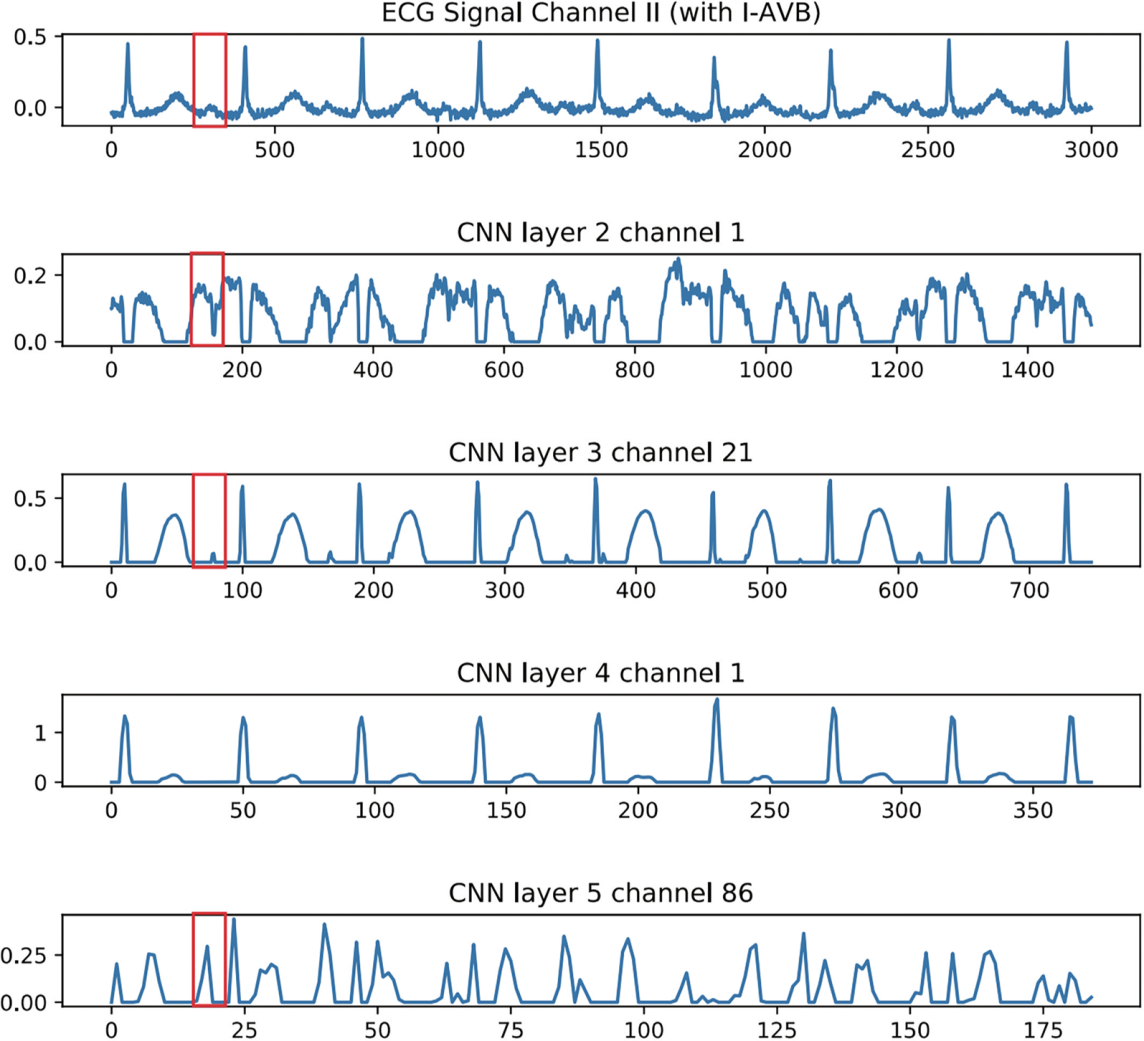
Features extracted by different CNN layers from ECG signals

Figure 7 shows that the embedding vectors of the normal and STE classes are very similar, the T waveforms of which are morphologically difficult to distinguish. This leads to the confusion between the two classes. Similarly, the embedding vectors of classes STD and STE are very different and their T waveforms are quite different, which makes it very easy to distinguish classes STD and STE.

#### Effectiveness of Feature Extraction for CNN

The features extracted by the CNN from the ECG signal were visualized and are shown in Fig. 9. One sample of first-degree atrioventricular block (I-AVB) was chosen and the features output by different CNN layers from the sample. I-AVB is a disease of the electrical conduction system of the heart, which can be indicated on the ECG by a prolonged PR interval larger than 0.20 s [35]. The PR interval is marked by a red rectangle on the feature captured by different CNN layers in Fig. 9. It can be seen from the figure that most CNN layers can capture the PR interval when inputting an ECG signal of I-AVB. This proves that the CNN can effectively extract the features of heart disease from ECG signals for diagnosis.

From Fig. 9, we can see that most CNN layers can capture the PR interval when input a ECG signal of I-AVB. This prove that CNN can effectively extract the feature of heart disease from ECG signals for diagnosis.

## Conclusions

An end-to-end model combining a CNN and transformer to classify ECG signals is proposed in this paper. In the model, a window function is employed to divide the ECG signal into different numbers of ECG segments. The feature information extracted by the CNN still has temporal characteristics. The combination of the CNN and improved transformer finally achieved an F1 score of 78.6%, which can be of great assistance to doctors or cardiologists [25]. In the future, our focus will be on the identification of more types of heart disease, such as myocardial infarction. It is hoped that the proposed model can be applied to low-cost ECG devices to facilitate diagnosis of heart disease in areas in medically underserved areas.

## Data Availability

The datasets used and analyzed during the current study are available from the corresponding author on reasonable request.
